# Rare co-occurrence of gastrointestinal stromal tumors and leiomyomas: A case report and review of the literature

**DOI:** 10.3892/etm.2026.13062

**Published:** 2026-01-09

**Authors:** Yu Zhang, Xiaoqiong Shi, Wenfu Xie, Meilin He, Shan Zhang, Xue Peng, Hao Lei, Xulei Li, Haiying Liu, Qiao Shu, Fangyuan Zou, Mingyong Wei

**Affiliations:** Department of Gastroenterology, Sichuan Science City Hospital, Mianyang, Sichuan 621000, P.R. China

**Keywords:** gastrointestinal stromal tumor, leiomyoma, gene mutation, co-occurrence

## Abstract

Gastrointestinal stromal tumors (GISTs) are recognized as the most common neoplasms originating from gastrointestinal mesenchymal tissue. On the other hand, gastric leiomyomas are a common benign neoplasm within the gastrointestinal tract. In general, GISTs are thought to be unrelated to gastric leiomyomas; however, the findings presented in the current study disprove this. The current case report presents a rare case where both GIST and gastric leiomyoma coexisted in a single subepithelial lesion (SEL) at the gastric cardia. Previous studies have confirmed that GIST can originate from smooth muscle cells with a BRAF*^V600E^* mutation, which was detected in the present GIST specimen. Notably, simultaneous expression of the KIT and BRAF*^V600E^* gene was also observed, challenging the previous assumption that only wild-type GIST would carry the BRAF*^V600E^* gene. In conclusion, it is proposed that there may be homology between GISTs and gastric leiomyomas, and the BRAF*^V600E^* mutation was the critical trigger. Therefore, the incidence of BRAF*^V600E^* might have been underestimated in reality. The present study highlights that clinicians should be aware that GIST and gastric leiomyoma can coexist in the same SEL to avoid misdiagnosis and mistreatment. In the face of the escalating drug resistance rate of GIST, researchers may derive some novel insights from the present findings for GIST treatment and management.

## Introduction

Gastrointestinal stromal tumors (GISTs) are rare in the context of all tumor types, with the incidence of GIST varying among different countries; however, there has been a gradual escalating trend over time (1-3). The global average incidence of GIST ranges from 1-2 per 100,000 person-years (4), whereas in China, it is ~0.40 per 100,000 person-years (5). In addition, GIST is the most common neoplasm originating from the gastrointestinal mesenchymal tissue (6), most commonly in the stomach, followed by the small intestine, colon, rectum and esophagus (7); the incidence of GIST at the esophagogastric junction is <1% (8). In previous years, the majority of GIST cases were initially misdiagnosed as leiomyoma or leiomyosarcoma, neurofibroma or neurilemmoma (4,9), and it was not until KIT (CD117), an immune marker specific to GIST, was discovered in the early 2000s, that GIST could be accurately diagnosed (9).

GISTs are typically identified during gastroscopy or computed tomography (CT) scan and usually appear as subepithelial lesions (SELs) on gastroscopy. SEL is a protrusion formation of the gastrointestinal tract (10), which typically originates from the deeper mucosa, being covered by epithelium, including GISTs, leiomyomas, neurilemmomas and lipomas (11). Leiomyoma is most common in the esophagus (12,13), and gastric leiomyoma is relatively uncommon; however, when present, it is most likely to occur in the cardia (14). Typically, GISTs and leiomyomas are viewed as different tumor categories that demonstrate no significant relationship.; however, the present study provided evidence that they can coexist.

The present report describes the rare co-occurrence of GIST and gastric leiomyoma in the same SEL at the cardia, and summarizes the clinicopathological and gene mutation characteristics, offering new insights and potential experience for the diagnosis and management of this condition. To the best of our knowledge, this is the first report on both GIST and gastric leiomyoma occurring in the same SEL at the gastric cardia.

## Case report

**Figure 1:**
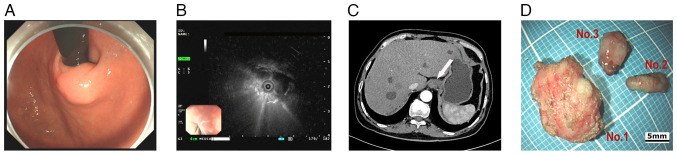
Clinical examination results and postoperative specimens. (A) Gastroscopy showed a subepithelial lesion at the cardia of the stomach. (B) Endoscopic ultrasonography described a heterogeneous hypoechoic mass with internal septa. (C) CT scan revealed slight homogeneous enhancement in arterial phase, no metastasis (the white arrow indicates the tumor). (D) After the operation, three solid tumors were obtained-gross appearance of tumors no. 1, 2 and 3 (scale bar, 5 mm).

A 72-year-old male patient with a history of type 2 diabetes, hypertension, acute pancreatitis and a pancreatic pseudocyst underwent a gastroscopy examination at Sichuan Science City Hospital (Mianyang, China) without presenting with any symptoms during a physical examination in June 2024. Physical examination showed mild upper abdominal tenderness, and the psychosocial and family medical histories of the patient were unremarkable. Gastroscopy identified chronic gastritis and a 15-mm SEL in the cardia (Fig. 1A); consequently, endoscopic ultrasonography (EUS) revealed the lesion originated from the muscularis propria, appearing as a heterogeneous hypoechoic mass with internal septa, which measured 13.9x9.2 mm (Fig. 1B). The CT scan showed a slightly thickened 15-mm nodule in the cardia with slight homogeneous enhancement and no signs of infiltration or metastasis (Fig. 1C).

**Figure 2:**
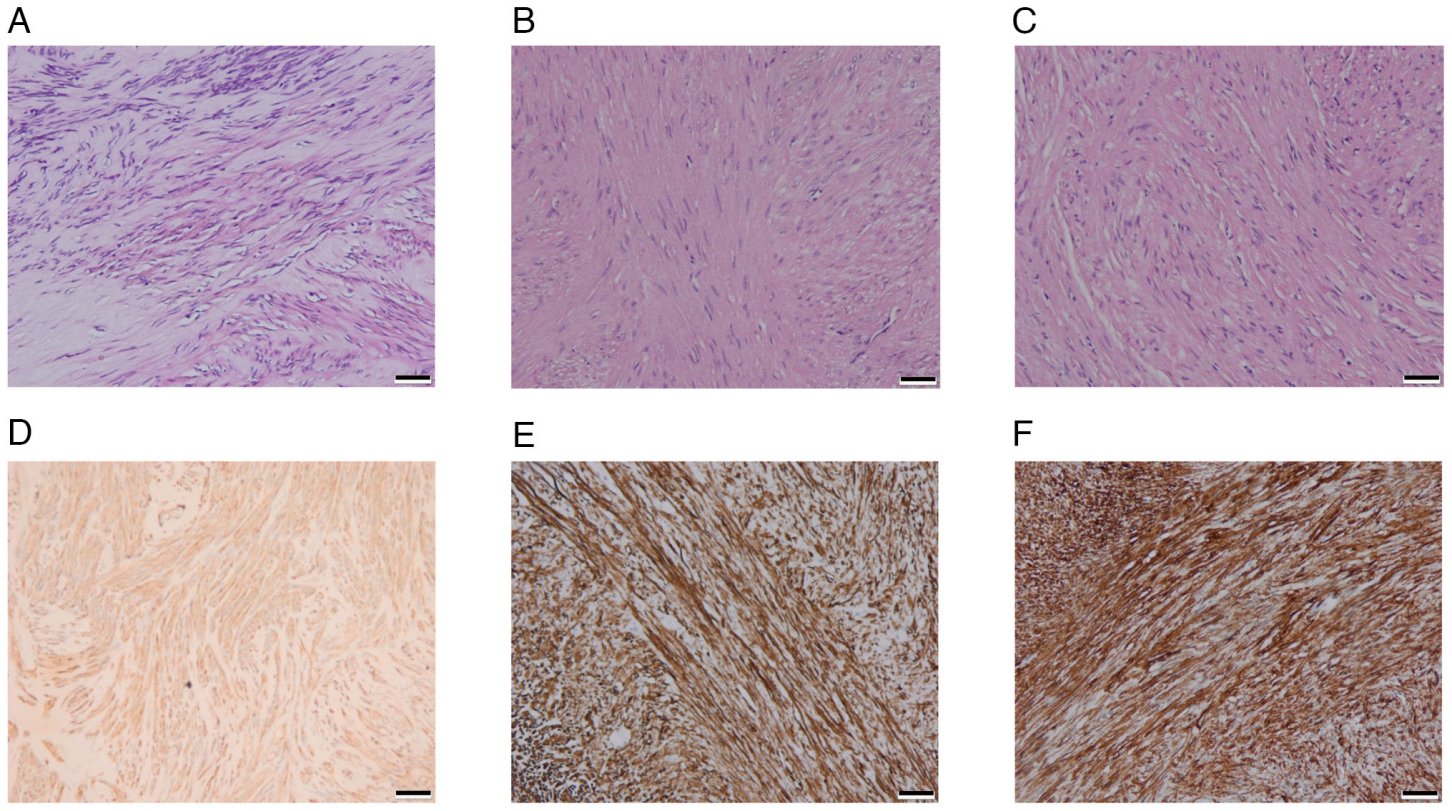
Histopathological and immunohistochemical images. Microscopically, (A) specimen No. 1 was composed of spindle-like cells with indistinct borders, and (B) the No. 2 and (C) No. 3 specimens exhibited spindle-like cells in bundles with defined borders (H&E staining; magnification, x20; scale bar, 50 µm). Immunohistochemically, (D) the No. 1 specimen was strongly positive for CD117 and a gastrointestinal stromal tumor, and (E) the No. 2 and (F) No. 3 specimens both were positive for Desmin, suggesting that they were leiomyomas (magnification, x20; scale bar, 50 µm).

An endoscopic submucosal dissection was performed to excise the lesion completely, and the patient fully recovered without complications. During the procedure, three solid tumors matching the EUS separation finding were removed; the three tumors were distinct with intact structures, light gray surfaces and moderate hardness (Fig. 1D). Tumor tissue specimens were fixed in 10% neutral buffered formalin at room temperature for 24-48 h. Following fixation, the tissues were embedded in paraffin and sectioned into 3-µm slices. The sections were then stained with hematoxylin and eosin (H&E) at room temperature, with hematoxylin applied for 5-10 min and eosin for 1-3 min. Histopathological analysis demonstrated that light-microscopically (H&E staining; x20 magnification), specimen No. 1 was composed of spindle-like cells with indistinct borders (Fig. 2A), while the No. 2 (Fig. 2B) and No. 3 (Fig. 2C) specimens had spindle-like cells in bundles with defined borders. The immunohistochemical (IHC) staining was performed on a fully automated IHC platform (Roche Diagnostics GmbH) according to the standardized protocol. Deparaffinization was carried out using the instrument s preconfigured settings. Heat-induced antigen retrieval was conducted with cell conditioner 1 reagent (Roche Diagnostics GmbH) in accordance with the manufacturer s protocol. Endogenous peroxidase activity was blocked by incubating the slides with 3% hydrogen peroxide blocking solution (Roche Diagnostics GmbH) at room temperature for 10 min. Primary antibody incubation was performed at 37˚C for 32 min using ready-to-use formulations. The antibodies applied included Pan-CytoKeratin (PCK) (cat. no. CCM-0960), CD34 (cat. no. CCM-0550), Desmin (cat. no. CDM-0023) and Ki-67 (cat. no. CKM-0032) (all from Fuzhou Maixin Biotech. Co., Ltd.); along with CD117 (cat. no. kit-0029), DOG-1 (cat. no. kit-0035), SOX-10 (cat. no. RMA-0726), and S-100 (cat. no. kit-0007) (all from Celnovte Biotechnology Co., Ltd.). Subsequently, slides were incubated with a secondary antibody detection system (cat. no. 760-500; Roche Diagnostics GmbH) at 37˚C for 8 min. All automated procedures were strictly executed following the instrument s pre-programmed protocol. Each staining run included both negative and positive controls. All sections were subsequently examined and analyzed under a light microscope.

Specimen No. 1 was histologically diagnosed as a GIST. According to the modified NIH criteria (15), the tumor had a mitotic count of 1/5 mm^2^ and was classified as G1 (low risk). IHC analysis revealed a profile positive for CD117, DOG-1 and CD34, and negative for PCK, Desmin, SOX-10 and S-100. The Ki-67 proliferation index was low at 1% (Fig. 2D).

**Figure 3:**
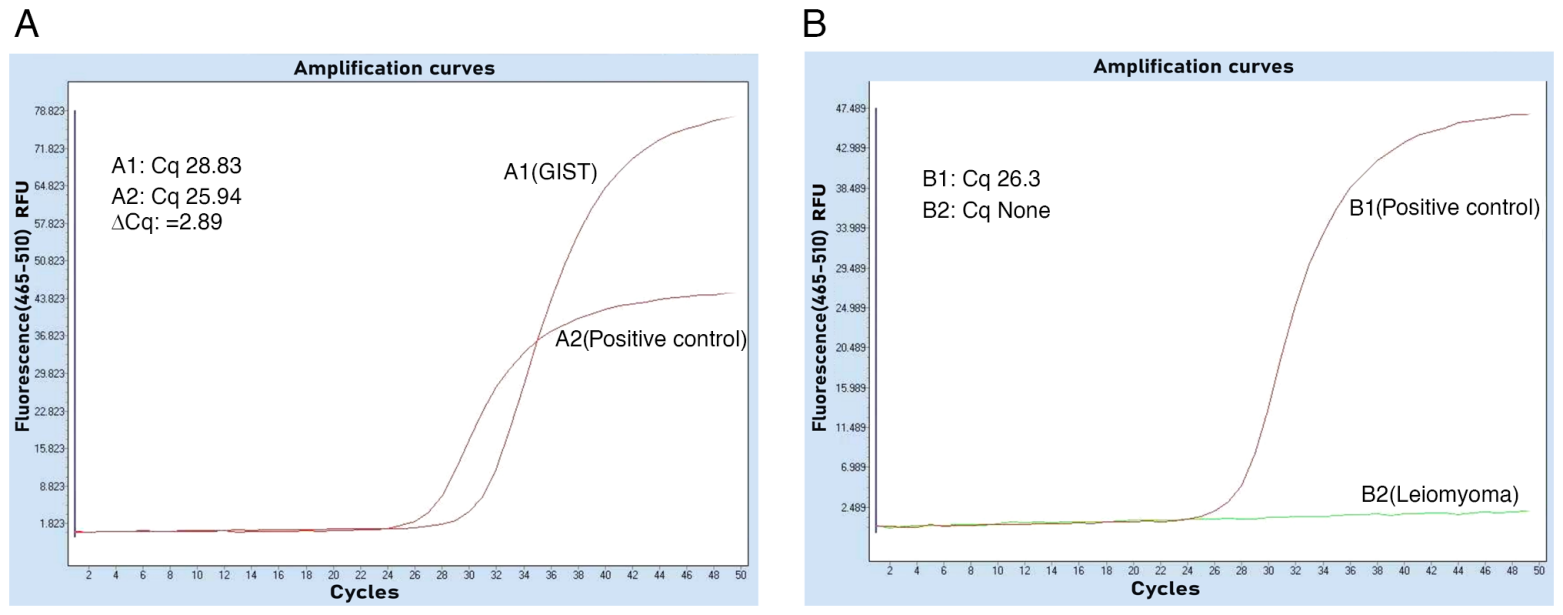
Gene testing by Roche Lightcycyler 480II fluorescent quantitative PCR. (A) Detection of BRAF*^V600E^* mutation was confirmed in the GIST, and the fluorescence on the y-axis is expressed in RFU. (B) No BRAF*^V600E^* mutations were identified in the leiomyoma. Cq, quantification cycle; ∆Cq, the difference between A1 and A2 in quantification cycle; None, no amplification of the target genes; GIST, gastrointestinal stromal tumor; RFU, relative fluorescence units.

Specimens No. 2 and No. 3 were leiomyomas with immunohistochemistry of PCK(-), CD117(-), Dog-1(-), CD34(-), Desmin(+), SOX-10(-), S-100(-) and Ki-67(+,1%) (Fig. 2E and F). The tumors underwent genetic testing through quantitative PCR, by the independent third-party laboratory, De-an Medical Testing Co., Ltd, using the LightCycler 480 II real-time PCR system (Roche Diagnostics). DNA was extracted from formalin-fixed, paraffin-embedded tissue specimens (obtained from the Sichuan Science City Hospital, Mianyang, China) by De-an Medical Testing Co., Ltd using the Nucleic Acid Extraction or Purification Reagent (Gene Tech Biotechnology), in strict accordance with the manufacturer s protocol. The procedure included deparaffinization, proteinase K digestion, decrosslinking, DNA capture on a filter membrane, two rapid wash steps and a final elution in the provided DNA elution buffer. For BRAF*^V600E^* mutation detection, quantitative PCR was performed using the Human BRAF Gene V600E Mutation Detection Kit (PCR-fluorescent probe method; Wuhan YZY Medical Science and Technology Co., Ltd) in strict adherence to the manufacturer s protocol. The qPCR assay was outsourced to De-an Medical Testing Co., Ltd. The thermal cycling conditions were as follows: Uracil-N-glycosylase treatment at 37˚C for 10 min; pre-denaturation at 95˚C for 5 min; followed by 40 cycles of denaturation at 95˚C for 15 sec and annealing/extension at 60˚C for 60 sec. The nucleotide sequences for the primers and probes were not provided by the manufacturer (cat. no. YZYMT-003; Wuhan YZY Medical Science and Technology Co., Ltd.). The results determined that a BRAF*^V600E^* mutation was present in the GIST, but not in the leiomyomas (Fig. 3A and B).

Based on established risk stratification guidelines, adjuvant targeted therapy was deemed unnecessary as an R0 resection had been achieved and the GIST was determined to be low risk (G1) (15-19). The management plan consisted of active surveillance with contrast-enhanced CT is typically performed every 6-12 months (15-19).

## Discussion

According to the current World Health Organization tumor classification, GIST is classified as a malignant tumor (20,21). The GIST primarily stems from Cajal cells or their precursor cells in the muscular connective tissue of the gastrointestinal tract (9), while a minor fraction may originate from smooth muscle cells (22). Cajal cells could be traced back to mesenchymal cells during the embryonic period (22). Gastrointestinal leiomyomas occur predominantly in the esophagus (12,13) and originate from the smooth muscle cells (23), which are also derived from embryonic mesenchymal cells (24).

Derived from the mesodermal germ layer, mesenchymal stem cells (MSCs) serve as multipotent precursors that generate the diverse spectrum of mesenchymal cells. This differentiation cascade culminates in the formation of mature bone, cartilage and connective tissue (25). Notably, interstitial cells of Cajal (ICC) are a specialized product of this cascade, originating directly from mesenchymal cells but whose developmental origin can be traced to the primordial MSC pool (26). Research by Radenkovic *et al* (27) indicates that mesenchymal cells give rise to c-KIT-positive precursors common to both ICC and smooth muscle cells. The divergent differentiation of this precursor pool is then directed by stem cell factor; precursors adjacent to ganglia commit to the ICC lineage, whereas the remainder differentiate into smooth muscle cells (27). Therefore, GIST and gastric leiomyoma originate from the same cellular lineages, suggesting a potential relation. Further research has revealed that GIST can develop from smooth muscle cells of the digestive tract by the BRAF*^V600E^* mutation (28-30). In a previous study, Kondo *et al* (31) successfully generated GIST cells by inducing a BRAF*^V600E^* mutation in smooth muscle cells using Myh11*^CreERT2^* and BRAF*^LSL-V600E/+^* mouse models. These findings provided the rationale for our detection into BRAF*^V600E^* mutation in patient. Notably, the BRAF*^V600E^* gene mutation was identified in the GIST specimen from the patient in the present study, thereby providing compelling evidence for the close association between GIST and smooth muscle cells. It would have been of interest to conduct single-cell sequencing on the present samples to elucidate the precise connection between GIST and gastric leiomyoma; however, at the time, this was not possible. Nevertheless, the co-occurrence of GIST and gastric leiomyomas within the same lesion provides some new insights for scientific exploration.

It is well known that most GISTs are positive for the gene KIT (CD117) (95%), DOG1 (>95%), CD34 (60-80%), α-smooth muscle actin (20-40%) and S-100 protein (5%). GISTs that are negative for the KIT gene occur in 5% of patients, and these exhibit epithelial cell morphology and a mutation in the platelet-derived growth factor receptor α (PDGFRA) gene (19). Numerous studies define some GISTs as ‘wild-type’ due to a lack of common driver mutations (such as KIT or PDGFRA); among these, BRAF*^V600E^* mutations represent a frequently observed alternative (32,33). The incidence of BRAF*^V600E^* mutations has been reported with a low frequency between 3.5 and 13.4% in wild-type GISTs, which is notably lower than KIT/PDGFRA mutations (30,34), leading to oversight frequently. In the clinical diagnosis of GIST, essential immunohistochemical tests include CD117, DOG1, actin, desmin, S-100 and CD34 (16,19). Therefore, physicians predominantly assess the KIT (CD117) and PDGFRA mutations without BRAF*^V600E^* in the diagnosis of GIST in clinical practice. However, the BRAF*^V600E^* mutations can co-occur with KIT. Consequently, the prevalence of BRAF*^V600E^* mutations in patients with GIST could be markedly underestimated. In addition, the present findings indicate that the KIT and BRAF*^V600E^* gene can be concomitant in a patient with GIST, which is consistent with other research. For example, during the investigation of BRAF*^V600E^* mutations in patients with wild-type GIST, researchers incidentally identified a case with co-existing KIT and BRAF*^V600E^* mutations in the same individual (34). This speculation is further corroborated in the research conducted by Jašek *et al* (35), in the present study, screening of 35 unselected GISTs revealed a significant finding, eight KIT/PDGFRA-positive tumors harbored concurrent BRAF*^V600E^* mutations. The co-occurrence involved five cases with KIT and three with PDGFRA mutations.

Most GISTs and leiomyomas are typically asymptomatic, and are frequently detected during gastrointestinal endoscopy (14,36). Early GISTs can be effectively treated through endoscopic or surgical resection, resulting in a favorable prognosis. The current guidelines in numerous countries advocate endoscopic resection for GISTs <2 cm, whereas for those >2 cm, the risk of metastasis escalates (17,19). The pathological results of GISTs determine whether the patient receives tyrosine kinase inhibitors (TKIs) (10,16,17,19); the accurate determination of genetic mutations plays a notable role in the diagnosis and treatment, as based on existing research, the efficacy of TKIs varies markedly among distinct gene mutation types (16,19). The current international guidelines advocate imatinib as the first-line treatment for patients with locally advanced unresectable and metastatic GIST, as well as those who have undergone complete resection with metastasis (15,19). The majority of GISTs harboring KIT mutations exhibit sensitivity to imatinib, whereas >50% of GISTs with PDGFRA mutations demonstrate resistance to imatinib, particularly those carrying the D842V mutation in exon 18 of PDGFRA (15,19). For patients with local GIST at high risk of recurrence, the standard regimen entails continuous imatinib for 3 years. The management of patients with metastases often requires a protracted course of drug therapy, unless there is an occurrence of intolerance or a specific request for discontinuation (18). However, the risk of TKI resistance is high due to the presence of primary multiple gene mutation mechanisms of GIST and subsequent secondary gene mutations during late-stage medication (37,38). Currently, there are no consensus guidelines for the treatment of GISTs harboring co-existing KIT and BRAF*^V600E^* mutations. In our view, for localized GIST <2 cm, endoscopic resection is the initial treatment of choice. For metastatic GIST, imatinib remains the first-line therapy. However, optimal treatment strategies for patients who develop resistance to imatinib require further investigation. The results of the current study present an innovative research direction in GIST treatment and management.

In conclusion, to the best of our knowledge, we propose for the first time that there may be homology between GISTs and gastric leiomyomas, and that the BRAF*^V600E^* mutation is the critical trigger; the incidence of BRAF*^V600E^* may have been underestimated by clinicians and researchers. Clinicians should recognize that GIST and gastric leiomyoma can coexist in the same SEL to avoid misdiagnosis and mistreatment. In the face of the escalating drug resistance rate of GIST, researchers may derive some novel insights from the present findings for GIST treatment and management.

## Data Availability

The data generated in the present study may be requested from the corresponding author.
